# Supplementary material for: Ifit1 regulates norovirus infection and enhances the interferon response in murine macrophage-like cells
Source: Wellcome Open Res. 2019 May 15;4:82. [Version 1] doi: 10.12688/wellcomeopenres.15223.1 (PMC6668250; doi:10.12688/wellcomeopenres.15223.1)
Supplement: Peer review history

# Peer review: Reviewer response for version 1

**DOI:** 10.21956/wellcomeopenres.16613.r35948

**Published:** 2019-07-29

**Authors:** Megan T. Baldridge

**Affiliations:** 1 Division of Infectious Diseases, Department of Medicine, Washington University School of Medicine, St. Louis, MO, USA

Mears *et al*. describe an analysis of the role of canonical ISG IFIT1 in regulation of murine norovirus infection in RAW264.7 cells. Enhanced MNV-1 viral titers were observed in 2 distinct cell lines lacking Ifit1, and the authors convincingly demonstrate that inhibition of viral replication by IFIT1 is independent of regulation of VPg-dependent translation. The authors suggest an alternate mechanism of regulation via promotion of type I IFN expression by IFIT1, though this is not explored in depth. Overall, this is a well-written manuscript, the data is robust, and this is an important contribution to the understanding of ISG-mediated control of norovirus infection.

 Comments:

1. It would be appropriate to reference the B cell model for human norovirus cultivation in addition to HIEs in the introduction ^1^.
2. In Figure 1A, IFIT1 is convincingly absent in KO1/2 lines. However, IFIT2 and IFIT3 levels also appear to be somewhat decreased. Can the authors please comment, especially as this may be related to the putative mechanism of suppression of IFN signaling in IFIT1 KO cells?
3. Could the authors please comment in figure legends on the number of experiments performed, and number of blots for which figures are representative? Are replicates within a single experiment or across multiple experiments?
4. The statement in the first paragraph of the discussion, "Ifit1 promotes type I IFN responses in RAW264.7 cells and as such contributes to the host antiviral response to restrict murine norovirus infection", is somewhat overstated, as the authors do not provide any direct evidence that IFN levels are differentially regulated by MNV in the presence or absence of Ifit1. The polyIC data is suggestive, but transfection of MNV RNA or analysis of IFN levels after infection at a variety of MOI would be helpful. If the authors can provide this data, this would be welcome addition to the study, or the conclusion should be softened.

I confirm that I have read this submission and believe that I have an appropriate level of expertise to confirm that it is of an acceptable scientific standard, however I have significant reservations, as outlined above.

## References

[1] : Enteric bacteria promote human and mouse norovirus infection of B cells. *Science*.2014;346(6210) : 10.1126/science.1257147 755-9 10.1126/science.1257147 25378626PMC4401463

# Peer review: Reviewer response for version 1

**DOI:** 10.21956/wellcomeopenres.16613.r35949

**Published:** 2019-07-22

**Authors:** Donna MacDuff

**Affiliations:** 1 Department of Microbiology and Immunology, University of Illinois at Chicago, Chicago, IL, USA

The manuscript from Mears *et al*., examines the role of murine Ifit1 as an antiviral factor for RNA viruses that utilize a VPg protein on the 5’ end of their RNAs in place of a cap structure. By generating two Ifit1 KO RAW264.7 (murine macrophage) cell lines, the authors show that Ifit1 suppresses murine norovirus (MNV) replication. Ifit1 has been shown previously to block the translation of capped mRNAs by interacting with the cellular translation machinery. However, using an in vitro translation assay, the authors demonstrate that IFIT1, as well as IFIT2 and IFIT3 do not inhibit translation of VPg-associated MNV, feline calicivirus (FCV) or porcine sapovirus (PSaV) RNAs. Cap0-associated RNAs are included as an important positive control for these assays. Evidence is also presented that, while IFIT1 can bind to Cap0-associated viral RNAs, IFIT1, IFIT2 or IFIT3 cannot bind to VPg-bound viral RNAs. Previous studies have shown that IFIT1 can regulate interferon (IFN) induction downstream of TLR3, TLR4 and MAVS. The authors confirm that IFIT1-deficiency impairs IFN induction after activation of intracellular RNA sensors by polyI:C transfection. However, they are unable to detect differences in IFN induction after stimulation of TLR3 or TLR4 with polyI:C or LPS treatment of WT and IFIT1 KO RAW cells. Overall, the experiments are well-controlled and the data are mostly convincing with multiple viruses tested. While the demonstration that IFIT1 controls MNV replication but cannot inhibit translation of VPg-RNAs is novel, the modulation of IFN induction by IFIT1 has been demonstrated by several other studies, and no further mechanistic insight is provided. The connection between IFIT1-mediated IFN induction after RNA sensing and regulation of MNV replication is inferred, but not demonstrated in this study.

 Major comments:

1. The authors conclude that IFIT1 modulation of the interferon response contributes to the restriction of MNV replication. However, no direct evidence is provided either that IFN induction after MNV infection is controlled by IFIT1, or that a defective IFN response is responsible for enhanced MNV replication in IFIT1 KO cells. Does IFNb treatment of cells restore control of MNV replication in IFIT1 KO cells? Since this study focuses of the anti-viral role of IFIT1 during calicivirus infection, it would be beneficial to measure IFN induction after MNV infection, particularly since the authors have shown previously that MNV-1 expresses an ORF that antagonizes the interferon response. Alternatively, the authors could revise this conclusion.
2. The figure legends state that statistical significance was assessed by t-tests. However, t-tests can only be used to compare two groups. One-way ANOVA is required for three groups (1WT and 2KO).

Minor comments:

1. The authors should clarify the nature of the WT RAW cells. Are they a polyclonal population, or a single clone derived at the same time as the two KO clones? Individual clonal cell populations can behave very differently. Does complementation of the KO cells with IFIT1 restore control of MNV replication or interferon induction?
2. In Figure 1 B and C, the authors demonstrate that MNV replicates to higher titers within 6-8 hours post-infection. However, viral titers at the time of infection are not shown. Is viral binding and entry equal in WT and KO cells?
3. For Figure 3, the doses of polyI:C and LPS used do not appear to be well optimized. 2 μg polyI:C is a large amount to transfect into cells (is this per ml?). A lower dose (0.2 μg) may allow larger differences in IFN induction to be observed. The IFN response to polyI:C and LPS treatment appears to be too minimal to detect differences in induction between the WT and KO cells and to draw conclusions from the data. Larger doses may be required.
4. The authors state that IFIT1 is not detectably-expressed in basal conditions and is induced within a few hours. The authors should consider how the timing of IFIT1 induction, differences in IFN induction (in Figure 3A or during MNV infection, which may take longer), and differences in MNV replication (Figure 1B and C) fit into their model.
5. The description of the experiments in Figure 3 in the text state that protein was extracted for analysis, but the data are not presented.

I confirm that I have read this submission and believe that I have an appropriate level of expertise to confirm that it is of an acceptable scientific standard, however I have significant reservations, as outlined above.

# Peer review: Reviewer response for version 1

**DOI:** 10.21956/wellcomeopenres.16613.r35676

**Published:** 2019-06-21

**Authors:** Yogesh Karpe

**Affiliations:** 1 Virology Lab, Nanobioscience Group, Agharkar Research Institute, Pune, India

Authors Mears *et al*. in their manuscript have investigated the antiviral role of IFIT1/ISG56 in response to calicivirus infection. Authors have shown that Ifit1 knockdown promoted MNV replication in a macrophage cell line. Finally, the authors demonstrated that Ifit1 knockdown cells have impaired cytoplasmic dsRNA sensing and weak type I interferon response. Overall this is a well-written manuscript containing results which merit indexing.

 Specific comments:

1. Comments should be made on the relevance of RAW264.7 cells for MNV infection studies. Why authors' selected this particular cells for their studies?
2. In this work, authors have purified recombinant Ifit1, Ifit2, and Ifit3. These recombinant proteins were used for *in-vitro* translation and primer extension inhibition assays. So please indicate the purity of the recombinant proteins used for the assay. Also please include the gel images of purified recombinant proteins in the manuscript.

I confirm that I have read this submission and believe that I have an appropriate level of expertise to confirm that it is of an acceptable scientific standard.
